# Differences in peak oxygen uptake in heart failure patients with and without cachexia: A systematic review and meta‐analysis

**DOI:** 10.14814/phy2.70663

**Published:** 2025-11-16

**Authors:** Bharathi Upadhya, Christoforos D. Giannaki, Pinelopi S. Stavrinou, Julee McDonagh, Mathias Schlögl, Gregory Y. H. Lip, Konstantinos Prokopidis

**Affiliations:** ^1^ Division of Cardiology, Department of Medicine Duke University School of Medicine Durham North Carolina USA; ^2^ Department of Life and Health Sciences University of Nicosia Nicosia Cyprus; ^3^ School of Nursing, Faculty of Science, Medicine & Health The University of Wollongong Wollongong New South Wales Australia; ^4^ Centre for Chronic and Complex Care Research Blacktown Hospital, Western Sydney Local Health District Blacktown New South Wales Australia; ^5^ Clinic Barmelweid AG Barmelweid Switzerland; ^6^ Danish Center for Clinical Health Services Research, Department of Clinical Medicine Aalborg University Aalborg Denmark; ^7^ Liverpool Centre for Cardiovascular Science at University of Liverpool, Liverpool John Moores University and Liverpool Heart and Chest Hospital Liverpool UK; ^8^ Department of Musculoskeletal Ageing and Science, Institute of Life Course and Medical Sciences University of Liverpool Liverpool UK

**Keywords:** cachexia, heart failure, physical function, rehabilitation, VO_2peak_

## Abstract

Chronic heart failure (CHF) is characterized by reduced peak oxygen consumption (VO_2peak_). Cachexia may exacerbate the decline in VO_2peak_ from reductions in muscle mass and strength. We sought to assess differences in VO_2peak_ between patients with CHF and cachexia and those without. A systematic literature search of cohort studies via databases (PubMed, Web of Science, Scopus, and Cochrane Library) was conducted from inception until April 2025. A meta‐analysis using a random‐effects model was employed. Overall, 10 articles were included in this study. There was a statistically significant reduction of mean VO_2peak_ in patients with CHF and cachexia versus those without cachexia (*k* = 10; MD: −2.21 mL/kg/min, 95%confidence interval [CI]: −2.95 to −1.47, *I* ^2^ = 51%, *p* < 0.01). When cachexia was defined as weight loss of ≥7.5% over the last 6 months, results remained identical (*k* = 6; MD: −2.47, 95% CI: −2.92 to −2.01, *I* ^2^ = 11%, *p* < 0.01). Meta‐regression analyses regarding age, sex, body mass index, and left ventricular ejection fraction showed no impact as potential moderators, and no publication bias was detected (*p* > 0.05). CHF patients with cachexia exhibit significantly decreased VO_2peak_ compared to their free‐cachexia counterparts.

## 1 INTRODUCTION

Peak oxygen uptake (typically reported as VO_2peak_) is a fundamental measure of cardiorespiratory fitness and, thus, an essential indicator of cardiovascular (CV) performance and health (Hawkins et al., 2007). This is the highest amount of oxygen one can utilize at peak exercise and is directly affected by the ability of the CV system to keep up with the oxygen delivery and the ability of the skeletal muscle (SM) unit to extract oxygen for its energy needs (Hawkins et al., 2007). VO_2peak_ is also a powerful predictor of CV mortality and morbidity (Laukkanen et al., 2004; Myers et al., 2002).

According to the Fick equation, VO_2_ equals the product of cardiac output (CO) and arterial–venous oxygen content difference (A‐VO_2_ Diff). This results in the following Fick equation: VO_2_ = CO [heart rate (HR) × stroke volume (SV)] × A − VO_2_Diff. Thus, VO_2peak_ may be affected by a ventilatory reserve (ability to pick up oxygen in the lungs), CV, and HR reserve (ability to increase SV and HR), peripheral vascular function, and SM abnormalities that result in reduced convective and diffusive O_2_ transport coupled with decreased O_2_ utilization by exercising muscle (Haykowsky et al., 2012; Houstis et al., 2018; Middlekauff, 2010; Phillips et al., 2015; Poole & Richardson, 1997; Tucker et al., 2022).

Chronic heart failure (CHF) is a systemic syndrome that leads to a cascade of physiological changes characterized by impaired exercise capacity and fatigue (Del Buono et al., 2019). A hallmark feature of CHF is reduced exercise tolerance, objectively measured by VO_2peak_ (Del Buono et al., 2019). VO_2peak_ is a valuable exercise capacity index and a strong prognostic marker in patients with CHF (Mancini et al., 1991). While impaired cardiac reserve is considered to be central in HF, reduced exercise and functional capacity result from multisystem dysfunction, including aging, impaired pulmonary reserve, as well as peripheral vascular and SM dysfunction in patients with HF (Del Buono et al., 2019).

SM mass is a major determinant of metabolic rate and oxygen consumption during exercise (Del Buono et al., 2019; Houstis et al., 2018; Middlekauff, 2010; Poole & Richardson, 1997; Tucker et al., 2022; Weiss et al., 2017). A variety of SM abnormalities develop in patients with CHF (Bekfani et al., 2020; Middlekauff, 2010; Weiss et al., 2017). Physical inactivity, commonly observed in CHF patients, leads to deconditioning, characterized by a reduction in SM mass and strength, further impairing the CV system's ability to support physical activity. Aging with CHF results in a systemic proinflammatory state, further accelerating SM mass loss (Krysztofiak et al., 2020). Therefore, reduced edema‐free muscle mass, as seen in conditions like cachexia, can decrease oxygen uptake and utilization, lowering VO_2peak_ (Harrington et al., 1997). Cachexia, a complex metabolic syndrome with underlying illness, is characterized by loss of muscle with or without loss of fat mass. It is associated with muscle wasting and altered metabolic and biochemical pathways, and has emerged as a significant determinant in the prognosis of HF. It is primarily defined as a loss of body mass (taking fluid imbalance into account) of more than 5% in 12 months or (bodymass index [BMI <20 kg/m^2^]) with accompanying conditions like decreased muscle strength, fatigue, anorexia, low fat‐free mass index and abnormalities in blood biomarkers (elevated C‐reactive protein and/or elevated interleukin‐6, Hemoglobin <12 g/dL, or low serum albumin (<3.2 g/dL)) (Anker & Morley, 2015; Loncar et al., 2016; von Haehling, 2017).

In CHF patients, cachexia may exacerbate the decline in VO_2peak_ by further reducing SM mass and strength, leading to a vicious cycle of decreased physical activity and worsening CV function. Nevertheless, it should be noted that, to date, there is no published systematic review or meta‐analysis that could provide an answer to the question of whether CHF patients with cachexia exhibit different levels of VO_2peak_ compared to patients with their free cachexia counterparts. The current systematic review and meta‐analysis aimed to investigate whether there are differences in VO_2peak_ between patients with CHF who have cachexia and those without cachexia.

## 2 METHODS

The revised 2020 Preferred Reporting Items for Systematic Reviews and Meta‐Analyses (PRISMA) criteria were followed to perform this systematic review and meta‐analysis (Page et al., 2021). The protocol is registered in the International Prospective Register of Systematic Reviews (PROSPERO) (CRD42024554057). A PRISMA checklist is provided in the Supplementary File.

### 2.1 Search strategy

From inception to April 2025, two investigators independently searched four databases (PubMed, Scopus, Web of Science, and Cochrane Library). The search phrases “heart failure AND cache*” were used in the search strategy. As part of the study, the PICO framework of “heart failure with cachexia,” “heart failure without cachexia,” “maximal oxygen capacity,” “peak oxygen uptake” was utilized. We only included human studies involving adult participants in our literature search. In addition, the institutional records were manually searched for available theses using the expertise of a medical librarian. A detailed description of the keyword search strategy is displayed in Table S1.

### 2.2 Study selection and outcomes of interest

#### 2.2.1 Study selection

Independent screening of title, abstract, and full text was performed by two reviewers who selected studies that met the inclusion criteria. Discussions occurred between the two reviewers, and disagreements were resolved by discussing with a third reviewer when required. Studies that could not be obtained in a full‐text English version were excluded.

#### 2.2.2 Types of studies included

We evaluated all prospective intervention studies, including parallel group trials (randomized and nonrandomized trials) with an intervention and a control group, as well as pre‐ and post‐intervention studies.

### 2.3 Participants

#### 2.3.1 Inclusion criteria

Adult patients with a diagnosis of CHF, irrespective of type, severity, and clinical setting (i.e., outpatients and inpatients), with age ≥18 years. We included and pooled results for patients with CHF, as the magnitude and implications of HF on VO_2peak_ are considered relatively consistent between HF phenotypes (Del Buono et al., 2019). In addition, neither left ventricular ejection fraction (LVEF) nor New York heart association functional class is directly associated with cachexia development (Anker et al., 1997).

### 2.4 Diagnosed with cachexia irrespective of definition

#### 2.4.1 Outcomes

To investigate whether there are differences in VO_2peak_ between patients with CHF who have cachexia and those without cachexia. The VO_2peak_ indexed to body mass is used here.

### 2.5 Data extraction and risk of bias

Two investigators extracted data independently, including the name of the first author, publication date, country of origin, participant age, sex, BMI, study design, VO_2peak_ values, and the method of assessment, cachexia definition, reported comorbidities, and LVEF. The Newcastle‐Ottawa Scale (NOS) was utilized to assess the quality of the included studies. NOS assigns a maximum of 9 points based on three quality parameters: selection, comparability, and outcome. Two investigators made the evaluation, and a third team member resolved any disagreements. The risk of bias will be consequently categorized as high (5 or fewer points), moderate (6, 7), or low (8, 9). For cross‐sectional studies, it was classified as high (≤3 points), moderate (4–5 points), or low (6–7 points) (Luchini et al., 2017). For randomized controlled trials (RCTs), the quality of the studies was evaluated using the risk‐of‐bias 2 (RoB2) tool (Sterne et al., 2019). RoB2 assesses bias according to five domains: (i) randomization process, (ii) deviations from intended interventions, (iii) missing outcome data, (iv) measurement of the outcome, and (v) selection of the reported result. Its scoring system defined bias as “high,” “some concerns,” or “low.”

### 2.6 Statistical analysis

Quantitative data were treated as continuous values using mean differences (MDs), while a random‐effects model and an inverse‐variance approach were used to determine statistical significance. Statistical heterogeneity of outcomes across studies was estimated using the overlap of their 95% confidence intervals (95% CI) and expressed as Cochran's *Q* (chi‐squared test) and *I* ^2^ measurements. Low heterogeneity was defined as *I* ^2^ between 30% and 49%, moderate heterogeneity between 50% and 74%, and high heterogeneity as 75% and above (Higgins et al., 2003). When substantial heterogeneity was present, a meta‐regression was performed to investigate potential sources of variability that could affect estimate rates across studies (Cumpston et al., 2019), including factors such as age, BMI, and LVEF. In case studies reported interquartile ranges (IQR), the formula “standard deviation (SD) = width of IQR/1.35” was used to estimate the missing SDs (Hozo et al., 2005).

Subgroup analysis was conducted based on a definition of weight loss of at least 7.5% during the last 6 months and other definitions. In addition, a sensitivity analysis was performed to assess the robustness of reported statistical results by controlling for the increased risk of bias of any included studies. The meta‐analysis was synthesized using Review Manager (RevMan 5.4.1) software. A *p*‐value of <0.05 was considered statistically significant. With at least 10 studies, we assessed the risk of publication bias in our analysis using Egger's weighted regression test and visually inspected funnel plots (Lin & Chu, 2018).

## 3 RESULTS

**FIGURE 1:**
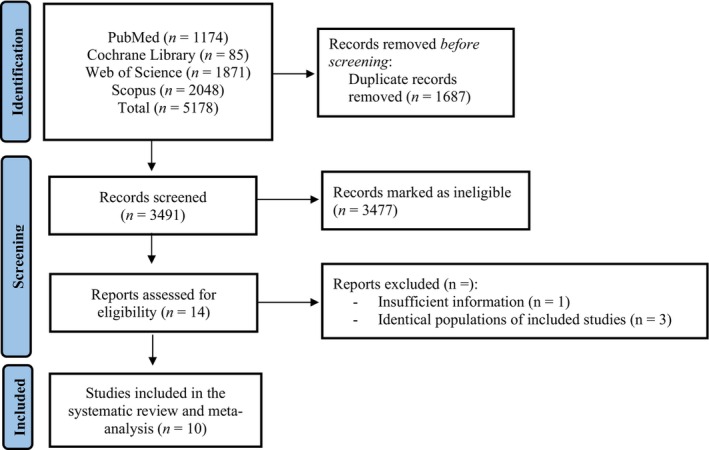
Flowchart of the search strategy.

The initial literature search yielded 5178 publications. Following the exclusion of duplicates, 3491 full texts were screened. Of these 3491 articles, 3477 were deemed ineligible, leaving 14 potential studies for inclusion. From these, three studies included participants as part of another study in our analysis (Sandek et al., 2024; Szabo et al., 2014; Valentova et al., 2016), while one did not provide sufficient information on VO_2peak_ data between those with cachexia and those without (Toth et al., 1997). Overall, 10 articles were included in this study. The PRISMA flow chart in Figure 1 summarizes the selection process.

### 3.1 Study population

From the available data collected, 15.8% of the sample consisted of females with cachexia and 17.5% without cachexia. The mean age of the population ranged between 57.7 and 72.7 years for those with cachexia and 56.0 and 72.0 years for those without cachexia. Mean LVEF for those with cachexia ranged from 23.3% to 33.6% and 21.8% to 38.7% for those without cachexia. Mean VO_2peak_ ranged from 9.8 to 17.9 mL/kg/min in patients with cachexia and 10.16 to 18.8 mL/kg/min in those without cachexia. In one study, patients with cachexia had a higher prevalence of sleep disorders and Cheyne–Stokes respiration (Hagenah et al., 2010). One study (Witte et al., 2008) examined respiratory exchange ratio without differences between groups, while in the two studies that examined VE/VCO_2_ slope (Witte et al., 2008, Davos et al., 2003), one study found a significantly higher slope in those with cachexia versus patients without (46.1 ± 15.2 vs. 36.1 ± 11.7) (Davos et al., 2003). Table 1 provides baseline clinical characteristics, VO_2peak_ assessment methods, and cachexia definition employed in each study.

**TABLE 1:** Study and participant characteristics of the included studies.

Study, year	VO_2_ assessment	Cachexia definition	Cachexia				Without cachexia			
			*n* (M/F)	Age	BMI	LVEF %	*n* (M/F)	Age	BMI	LVEF %
Hagenah et al. (2010)	‐	Non‐intentional, non‐oedematous, documented weight loss of more than 7.5% of their previous normal weight over a period of >6 months	12 (−)	64.8 (14.5)	25.2 (3.2)	23.3 (7)	13 (−)	57.3 (11.6)	26.4 (4)	21.8 (5)
McEntegart et al. (2007)	Symptom‐limited treadmill stress testing using the STEEP (Standardized Exponential Exercise Protocol) protocol	Non‐intentional weight loss of >7.5% body weight over 6 months	10 (8/2)	72.7	20 (5)	27.1 (11.9)	20 (18/2)	67.6	29.2 (4.2)	35.4 (9.7)
Filippatos et al. (2003)	Weber protocol	Non‐intentional non‐edematous weight loss of more than 7.5% of the premorbid weight	8 (−)	63.1 (8)	‐	26 (5)	13 (−)	60.4 (9.9)	‐	26 (5)
Davos et al. (2003)	Bruce protocol	Non‐intentional non‐edematous weight loss of more than 7.5% over the last 6 months	64 (60/4)	65.4 (12)	27.5 (4.3)	26.3 (15.1)	525 (434/91)	61 (12.4)	21.5 (2.5)	31.6 (14.8)
Nagaya et al. (2001)	‐	Non‐intentional non‐edematous weight loss of more than 7.5% over the last 6 months	28 (17/11)	60 (15.9)	17.9 (2.1)	27 (3.3)	46 (31/15)	61 (13.6)	22.7 (4.8)	29 (6.8)
Doehner et al. (2001)	Bruce protocol	Non‐intentional non‐edematous weight loss of more than 7.5% over the last 6 months	19 (19/0)	66 (13.1)	21.5 (1.7)	27 (17.4)	28 (28/0)	58 (10.6)	27.2 (3.7)	29 (15.9)
Sandek et al. (2014)	Modified Naughton protocol	Nonedematous unintentional weight loss of 5% or more within the previous 6 to 12 months	12 (4/8)	64 (9)	25 (5)	25 (7)	53 (36/17)	66 (9)	29 (5)	31 (7)
Moughrabi et al. (2023)	‐	Lost at least 6% of their baseline weight at 6 months	13 (11/2)	57.7 (15)	31.4 (5.7)	24.5 (9.5)	101 (67/34)	56 (12.8)	28.3 (4.7)	34.2 (12.2)
Witte et al. (2008)	‐	Documented history of significant weight loss over the preceding 6 months of >6%, and a BMI <25 kg/m^2^	9 (−)	72 (5)	22.2 (2.1)	33.6 (3)	5 (−)	72 (6)	32.9 (6.7)	34.3 (3.3)
Emami et al. (2018)	Modified Bruce protocol	Presence of non‐oedematous, non‐intentional weight loss of ≥6% over a period of at least 1 year as per previously published definition	25 (25/0)	64.3 (13.4)	28.7 (4.3)	31.2 (9.7)	138 (138/0)	66.4 (10.8)	30.1 (4.8)	38.7 (12.8)

*Note*: Data are expressed as mean (standard deviation).

Abbreviations: BMI, body mass index; LVEF, left ventricular ejection fraction.

### 3.2 VO_2peak_ in chronic heart failure with cachexia versus without cachexia

**FIGURE 2:**
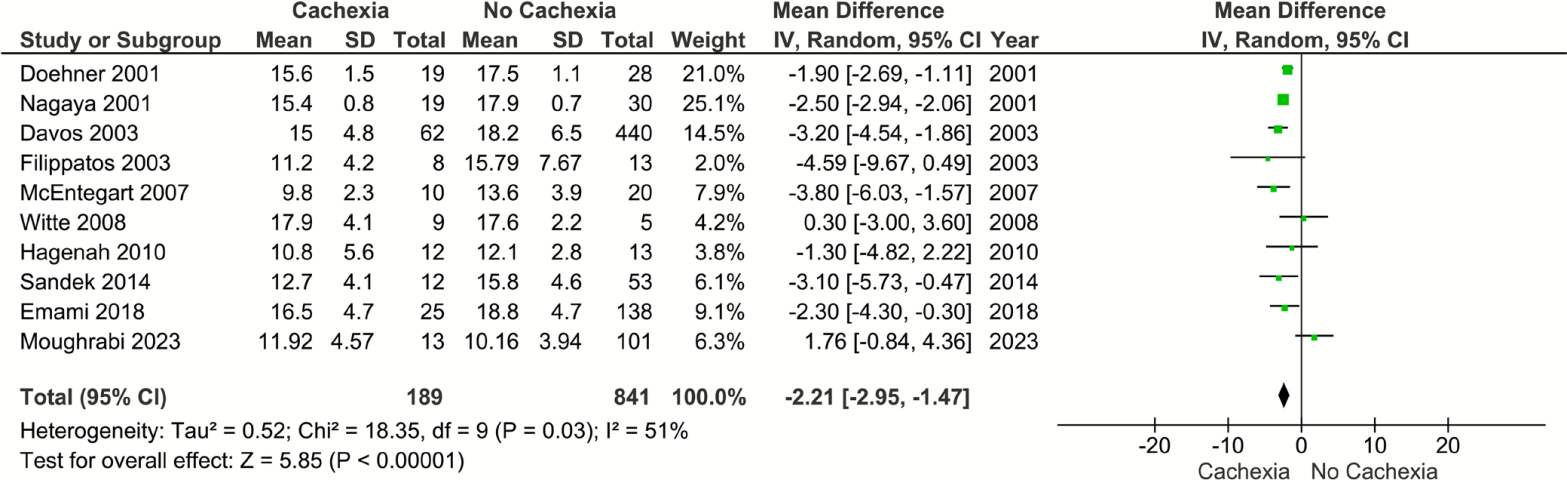
Differences in VO_2peak_ in patients with heart failure with versus without cachexia.

**FIGURE 3:**
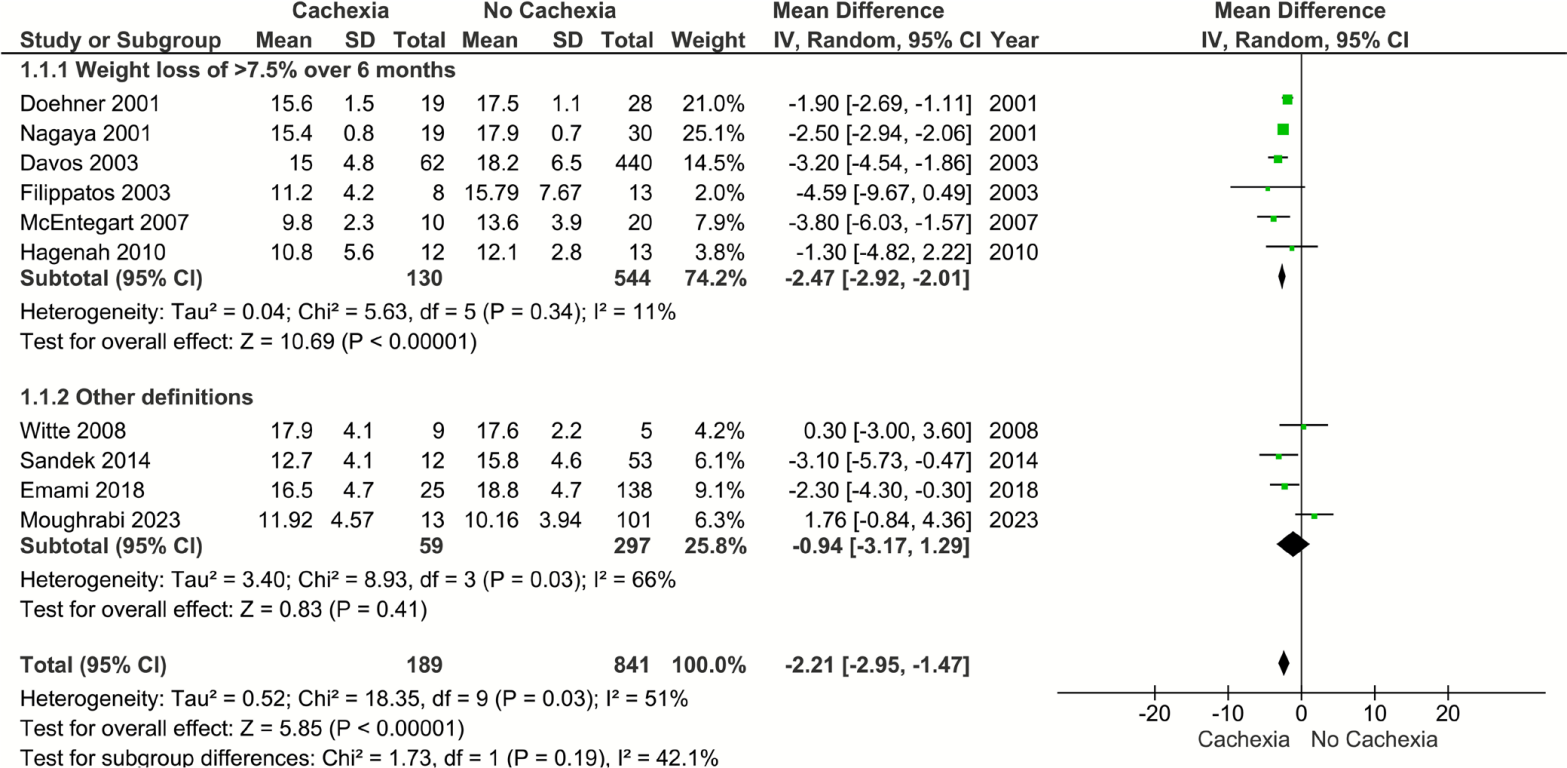
Differences in VO_2peak_ in patients with heart failure with versus without cachexia, where cachexia was defined as weight loss of ≥7.5% over the last 6 months.

Our main analysis demonstrated a statistically significant reduction of mean VO_2peak_ in patients with CHF and cachexia versus those without cachexia (*k* = 10; MD: −2.21 mL/kg/min, 95% CI: −2.95 to −1.47, *I* ^2^ = 51%, *p* < 0.01) (Figure 2). Considering the variation of cachexia definition, we performed a sensitivity analysis, for which we calculated the effect sizes only for studies in which cachexia was defined as weight loss of ≥7.5% over the last 6 months. However, results remained identical (*k* = 6; MD: −2.47, 95% CI: −2.92 to −2.01, *I* ^2^ = 11%, *p* < 0.01) (Figure 3).

### 3.3 Meta‐regression analyses and publication bias

Meta‐regression analyses regarding age, BMI, LVEF, and sex showed no impact as potential moderators (*p* > 0.05) (Table S2). Additionally, no publication bias was detected (*p* = 0.12) (Table S3).

## 4 DISCUSSION

This systematic review and meta‐analysis revealed a significant reduction of mean VO_2peak_ in patients with CHF and cachexia versus those without cachexia. Results remained the same with a sensitivity analysis, for which we calculated the effect sizes only for studies in which cachexia was defined as weight loss of ≥7.5% over the last 6 months. Meta‐regression analyses regarding age, sex, BMI, and LVEF showed no impact as potential moderators. Additionally, no publication bias was detected. As far as we know, this is the first published systematic review or meta‐analysis to reveal that patients with CHF and cachexia exhibit significantly reduced VO_2peak_ compared to patients with their free cachexia counterparts. Our study showed the importance of cachexia in CHF patients and its association with marked exercise intolerance and urged physicians to detect and treat it in the early stages of CHF.

### 4.1 Interconnections between cachexia and chronic heart failure

Using different definitions, the prevalence of cachexia has been found to range from 5% to 20% in chronic HF (Ebner et al., 2013) Cachexia is a complex, severe, multifactorial condition, and it is an important predictor of poor clinical prognosis, reduced survival, and elevated hospital costs (Anker et al., 2003; Goyal et al., 2016). Malnutrition, systemic nutritional deficiencies, elevated inflammatory cytokines, immune system hyperactivity, and neurohormonal alterations accompany both cachexia and CHF (Lena et al., 2018; Soto et al., 2022; von Haehling et al., 2017). In addition, chronic HF is a systemic syndrome associated with a catabolic state that may lead to cachexia when the physiological balance between anabolic and catabolic processes is impaired, resulting in compensatory proteolysis and subsequent SM mass (von Haehling et al., 2017; Yoshida et al., 2013). Aging, along with multiple comorbidities, produces a proinflammatory state. It is another predisposing factor for the development of both cachexia and CHF. Due to a common pathophysiological pathway, patients with CHF often suffer from a frailty syndrome with sarcopenia as a component and cardiac cachexia as the last stage (Soto et al., 2022). Muscle wasting is an important component of cachexia (Loncar et al., 2016). SM loss often precedes cachexia development and can also predict poor results in HF.

### 4.2 Role of skeletal muscle in the pathophysiology of chronic heart failure

VO_2peak_ is a function of oxygen delivery (CO, Hemoglobin) and oxygen utilization (muscle mass and function) and is influenced by ventilation, cardiac, vascular, and SM mass and function. Thus, impaired SM mass and function play a significant role in reduced VO_2peak_. SM mass is an important determinant of exercise capacity in patients with CHF (Del Buono et al., 2019; Haykowsky et al., 2013; Houstis et al., 2018; Middlekauff, 2010; Sullivan & Hawthorne, 1995; Tucker et al., 2022; Weiss et al., 2017). Many biochemical, histological, and functional changes were seen in the SM of patients with CHF, and many of these abnormalities closely relate to exercise intolerance (Bekfani et al., 2020; Carbone et al., 2020; Harrington et al., 1997; Haykowsky et al., 2013; Houstis et al., 2018; Kitzman et al., 2017; Middlekauff, 2010; Upadhya & Kitzman, 2024; Weiss et al., 2017). SM abnormalities are likely caused by chronic inflammation, neurohormonal activation, and SM hypoperfusion and are intrinsic to the HF syndrome rather than being a secondary consequence or an epiphenomenon. In addition, physical inactivity, commonly observed in CHF patients due to symptoms such as fatigue and exercise intolerance, exacerbates the decline in VO_2peak_. This inactivity leads to deconditioning, characterized by a reduction in muscle mass and strength, further impairing the CV system's ability to support physical activity. Consequently, CHF patients often find it challenging to engage in even basic daily activities, leading to a diminished quality of life and increased dependency on others (Mapelli et al., 2023). Also, CHF is most commonly seen in older adults; along with aging, CHF results in a systemic proinflammatory state, which further accelerates the loss of muscle mass (Krysztofiak et al., 2020).

### 4.3 Potential therapeutic implications

After considering the relationship between cachexia, SM mass, and VO_2peak_, one might hypothesize that VO_2peak_ would increase after interventions are directed towards increasing SM mass and function. Annual screening tests for unintentional weight loss in CHF patients are needed. Seeking optimal biomarkers of cachexia to facilitate diagnosis is very important. Pre‐albumin seems to be a biomarker of cachexia, a potential indicator of malnutrition, low cholesterol levels, and worse prognosis in chronic HF patients (Araújo et al., 2011). SM function improvement after exercise training shows how physical activity is the primary treatment for these patients (Haykowsky et al., 2013). Physical exercise programs and nutritional supplementation are effective in individuals with low functional levels (Soto et al., 2022). The effects of other supplements, including essential amino acids, omega‐3 polyunsaturated fatty acids, or pharmacological agents like immunomodulators, anabolic hormones, appetite stimulants, and other new drugs, are currently under investigation (von Haehling & Anker, 2016). Randomized studies are needed to define the possible effects of these means on SM mass and, consequently, on functional capacity.

### 4.4 Strengths and limitations

Sources of heterogeneity, including age and BMI, were explored through meta‐regression, offering insights into moderating factors. Additionally, publication bias was observed for CO, suggesting caution in interpreting these findings. Moreover, medication count among studies may have been over‐ or underreported due to potential inaccuracies arising from errors in drug prescription coding or incorrect electronic tabulations. Furthermore, the small number of studies (*n* = 10) and underrepresentation of females (15.8%–17.5%) may limit generalizability, whereby some studies lacked detailed comorbidity data, affecting comparability between the two groups. Variations in CHF populations, such as differences in baseline weight, HF severity, comorbidities, and treatment regimens, as well as inconsistent methods for evaluating cachexia definition criteria components (e.g., muscle strength and fat‐free mass), can confound the results and limit our ability to isolate the specific impact of cachexia on V̇O_2_peak. However, this highlights the complex, multifaceted nature of cachexia in HF. Our study was prone to limitations about the cross‐sectional nature of the data utilized for analysis, the increased heterogeneity among studies, the increased risk of bias from multiple studies incorporated in our analyses, and the lack of studies allowing for distinction between reduced and preserved ejection fraction rates regarding the risk of cachexia. We did not have information about diet or patients' participation in exercise programs; the beneficial effect of these measures to prevent or treat cachexia has not been evaluated. Additionally, our studies included employed multivariate analysis, for which, the confounders used were not identical among studies, while the inability to stratify or adjust for HF stage (e.g., NYHA class) or key comorbidities due to incomplete reporting in primary studies is another limitation. Although meta‐regression by LVEF showed no significant moderation, residual confounding by disease severity or comorbid burden cannot be excluded.

## 5 CONCLUSION

CHF patients with cachexia exhibit significantly decreased VO_2peak_ compared to their free‐cachexia counterparts. A patient's nutritional status and cardiac cachexia require more attention from medical professionals, as these conditions are associated with a worse prognosis for CHF patients. Methods of prevention of cachexia and treatment are needed in patients with CHF.

## FUNDING INFORMATION

No funding was received for this manuscript.

## CONFLICT OF INTEREST STATEMENT

Authors declare no conflict of interest.

## ETHICS STATEMENT

None.

## Supporting information


**Table S1.** Meta‐regression analyses evaluating age, BMI, LVEF, and sex as potential moderators.

**Table S2.** Publication bias using Egger’s test.

**Table S3.** Risk of bias assessment of the included studies.

Data S1.

## Data Availability

Data is available upon request.
