# Supplementary material for: Differences in peak oxygen uptake in heart failure patients with and without cachexia: A systematic review and meta‐analysis
Source: Physiol Rep. 2025 Nov 16;13(22):e70663. doi: 10.14814/phy2.70663 (PMC12620407; doi:10.14814/phy2.70663)
Supplement: Supplementary file 1 — Table S1. Meta‐regression analyses evaluating age, BMI, LVEF, and sex as potential moderators. [file PHY2-13-e70663-s004.docx]

**Table S1.** Meta-regression analyses evaluating age, BMI, LVEF, and sex as potential moderators.

|  |  |  |  |  |
| --- | --- | --- | --- | --- |
| **VO_2_max** | *p* | t^2^ | SE | I^2^ |
| **Age** | 0.85 | 4.08 | 2.04 | 100% |
| **BMI** | 0.78 | 1.91 | 1.94 | 100% |
| **LVEF** | 0.71 | 2.01 | 2.01 | 100% |
| **Sex** | 0.62 | 2.17 | 2.99 | 100% |

BMI, body mass index; LVEF, left ventricular ejection fraction
