# Supplementary material for: Differences in peak oxygen uptake in heart failure patients with and without cachexia: A systematic review and meta‐analysis
Source: Physiol Rep. 2025 Nov 16;13(22):e70663. doi: 10.14814/phy2.70663 (PMC12620407; doi:10.14814/phy2.70663)
Supplement: Supplementary file 2 — Table S2. Publication bias using Egger’s test. [file PHY2-13-e70663-s003.docx]

**Table S2.** Publication bias using Egger’s test.

|  | ***p*** | ***t*** | ***b*** | **95%CI** |
| --- | --- | --- | --- | --- |
| VO_2_max | 0.12 | 1.7412 | -3.2151 | -3.65 – -2.78 |
